# Comparison of Agar Dilution, Broth Dilution, Cylinder Plate and Disk Diffusion Methods for Evaluation of Anti-leishmanial Drugs on *Leishmania* promastigotes

**Published:** 2012

**Authors:** T Mohammadzadeh, SM Sadjjadi, P Habibi, B Sarkari

**Affiliations:** 1 Department of Parasitology and Mycology, School of Medicine, Shiraz University of Medical Sciences, Shiraz, Iran; 2 Basic in Infectious Diseases Research center, Shiraz University of Medical Sciences, Shiraz, Iran

**Keywords:** Leishmaniasis, Drug Assay, Agar Dilution, Cylinder Plate, Disk Diffusion

## Abstract

**Background:**

Leishmaniases are a group of diseases caused by *Leishmania* parasites. Growing of drug unresponsiveness in leishmaniasis patients necessitates the development of new drugs and accordingly a suitable assay is needed for evaluation of any modalities. The aim of this study was to compare four drug assays methods, agar dilution, broth dilution, cylinder plate and disk diffusion, for evaluation of anti-leishmanial drugs on *Leishmania* promastigotes, using glucantime as a currently available drug for treatment of leishmaniasis.

**Methods:**

For broth dilution method, different concentration of glucantime was added to the parasite culture (promastigotes of *Leishmania*), while in cylinder plate method wells were punched in agar gel and filled with different concentration of drug and zone of inhibition was measured in each well. In disk diffusion method, the parasites were cultivated on the surface of agar; filter paper disks were enriched with various concentration of glucantime and were placed on the surface of agar. In agar dilution method, various concentrations of drug were incorporated onto blood agar and the parasites were cultivated on the surface of the agar.

**Results:**

A direct correlation was found between the drug concentration and size of inhibitory zones in cylinder plate and disk diffusion methods. These two drug assays methods provided much better performance in comparison with broth and agar dilution methods.

**Conclusion:**

Cylinder plate and disk diffusion methods seem to be acceptable methods for susceptibility testing of anti-leishmanial compounds on *Leishmania* promastigotes.

## Introduction

Leishmaniasis refers to a diverse group of diseases caused by different species of *Leishmania*. The disease pattern ranges from self-healing cutaneous lesions to debilitating muco-cutaneous ulcer, subclinical viscerotropic dissemination and fatal visceral involvement (1).

The leishmaniases are now endemic in 88 countries on five continents with a total of 350 million people at risk. It is believed that globally 12 million people are affected by leishmaniasis; this figure includes cases with overt disease and those with no apparent symptoms (2). Pentavalent antimonial compounds are the mainstay for treatment of leishmaniasis. Growing of antimony-resistant leishmaniasis patients in the last two decades, call for development of new drugs. Suitable assay is needed for development and evaluation of any new anti-*Leishmania* compounds.

Currently, evaluation of new drugs against leishmaniasis is performed on parasites, cultivated in liquid or biphasic media (3–5). These methods are usually time consuming, difficult to perform and the cultures can easily get contaminated with bacteria.

The present study was performed to compare four drug-evaluating assays, agar dilution, broth dilution, cylinder plate and disk diffusion methods for screening of anti-leishmanial compounds on *Leishmania* promastigotes.

## Materials and Methods

### Parasite

Promastigotes of *Leishmania major* (MRHO/IR/75/ER, from Pasteur Institute, Tehran) and *L. infantum* (isolated from a patients with visceral leishmaniasis (VL) and characterized in Department of Parasitology and Mycology, Shiraz, Iran) were cultivated in BHI medium (Merck, Germany) supplemented with 10% fetal calf serum (FCS).

### Broth dilution method

In broth dilution method, various concentrations (120, 60, 30, 15, 7.5, and 3.7 mg/ml) of glucantime (Sigma, USA) were inoculated with a standard suspension of *Leishmania* parasites (1×10^5^ promastigote/ml). After three days of incubation at 25°C, the parasites were counted and the MIC was determined by finding the lowest concentration of the drug that inhibited the growth of the parasite.

### Cylinder plate method

In this method, the blood agar was prepared in six cm plate by mixing BHI agar (Merck, Germany); BHI broth (Merck, Germany), distilled water, penicillin (240 U/ml), streptomycin (200 µg/ml) and 30 ml of rabbit blood. *Leishmania* parasites were cultivated onto the surface of agar plate. The gel was then punched to make 5-mm diameter holes. Each hole was filled with a different concentration of glucantime (120, 60, 30, 15, 7.5, 3.7 mg/ml). Following 3-5 days of incubation in candle jar at 25°C, the diameter of the zone of inhibition was measured for each well.

### Disk diffusion method

The agar was prepared as in cylinder plate in six cm plate and *Leishmania* parasites were cultivated onto the surface of the agar plate. Then, filter paper disks impregnated with different concentrations of drug, as mentioned before, were placed on the agar. After 3-5 days of incubation of the plates in candle jar at 25°C, the diameter of the zone of inhibition (ZI) of parasite growth around each disk was measured. The size of the ZI is inversely proportional to the MIC of the organism.

### Agar dilution method

Various concentrations of drug (as above) were incorporated onto blood agar. The parasites were cultivated on the surface of the agar. The plates were incubated in candle jar at 25°C for 3-5 days and checked by observing the lowest drug concentration that inhibited visible parasite growth. This concentration was reported as the MIC.

In all assays, the drug-free culture media was used as control and all assays were performed in duplicate, three times.

## Results

In broth dilution method, using *L. major* and *L. infantum*, no parasite growth was observed in concentration equal or above 15 mg/ml of drug while parasite growth was seen in drug concentration of 7.5 mg/ml and lower. Accordingly, MLC (Minimum Leishmanicidal Concentration) of drug for broth dilution method was found to be 15 mg/ml.

**Fig. 1:**
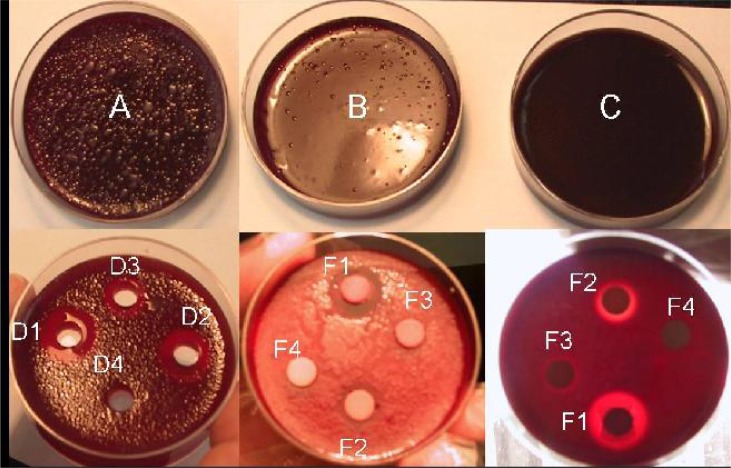
Comparison of agar dilution, cylinder plate and disk diffusion methods for evaluation of anti-leishmanial (glucantime) drug. A, B, C: 15, 30 and 60 mg/ml of glucantime in agar dilution method. D1, D2, D3: 60, 30, 15 mg/ml of glucantime and D4: control (distilled water) in cylinder plate method. F1, F2, F3: 60, 30, 15 mg/ml of glucantime and F4: control (distilled water) in disk diffusion method

In agar dilution method, both species of the parasite were survived and managed to grow in concentration up to 30 mg/ml of drug but parasite growth was not observed in concentration equal or above 60 mg/ml of drug (Fig.1).

Therefore, the MLC in this method was 60 mg/ml of drug concentration. The MLC for broth dilution and agar dilution were derived from duplicate assays performed three times for each sample.

In cylinder plate method, no zones of inhibition was obtained around the control wells on agar while in drug-containing wells inhibition zone was seen and their diameter was increased with increasing of drug concentrations (Fig.1) where a direct correlation was found between the drug concentration and size of inhibitory zones.

In addition, in disk diffusion method, no zone of inhibition was seen around the control disk, whereas in drug-containing disks, zones of inhibition were seen and their diameters were increased with increasing of drug concentration (Fig. 1).

## Discussion

Effective and inexpensive chemotherapeutic agents for the treatment of leishmaniasis are not available (6). Antimonial compounds like pentavalent antimonial drugs are the first-line treatment for this disease, whereas amphotericin B is being used as alternative drug. The clinical value of antimony therapy is now challenged because an increasing rate of treatment failure has been reported from different leishmaniasis endemic areas (7). Furthermore, amphotericin B has disadvantage of severe toxicity and high cost for a disease prevalent in third-world countries. Therefore, development of new drugs is urgently required for proper treatment of leishmaniasis. A variety of methods are available for evaluation of a new drug developed against various microorganisms. These are including agar dilution (8–10), disk diffusion (9, 11–13), cylinder plate (14) and broth dilution method (9–11).

The main stage of *Leishmania* parasite which is available in vertebrates, including human, is amastigotes. Therefore it makes sense that any drug assay for *Leishmania* be tested on this form of parasite. However, many currently available studies have been using promastigotes of *Leishmania* for evaluating of plant extracts or different antileishmanial compounds and in few studies a reasonable correlation has been found between the results of amastigote and promastigote assays. Here we compared four drug-evaluating assays, agar dilution, broth dilution, cylinder plate and disk diffusion methods on *Leishmania* promastigotes.

Broth dilution method is usually used for drug assay against *Leishmania* (4, 15–16). However this method is usually difficult to perform, is time consuming and the cultivated parasite can easily get contaminated with bacteria.

To circumvent this problem, cultivation of parasite on solid media has been developed (17). In this study, agar media supplemented with rabbit blood was used in three different systems; agar dilution, disk diffusion and cylinder plate and results were compared with broth dilution method.

Findings of this study showed that in broth dilution and agar dilution methods, parasite growth decreased with increasing of drug concentration but the MLC obtained in two methods were different. This might be due to cultivation of parasite on the surface of media in agar dilution method where the parasites might not be exposed to the whole drug. Furthermore, temperature might have a deteriorating effect on glucantime during agar preparation. It is worth mentioning that simultaneous testing of several isolates is possible with agar dilution method. However, this method is labor intensive and time consuming.

In cylinder plate method, inhibition zone was observed in wells from 10 mg/ml and higher of drug concentration and a direct correlation was found between drug concentration and size of inhibition zones. Same result was found with disk diffusion method where inhibition zone of growth was observed in disks with 10 mg/ml of drug concentration and a direct correlation was seen between size of inhibition zones and drug concentration.

Although, inhibition zone in both agar methods (cylinder plate and disk diffusion) was increasing with increasing of drug concentration, but diameters of zone was different in two methods. Absorption of drug by disk paper and reducing the diffusion of drug to the surface of agar plate might be accounted for these differences.

Comparison of the results obtained by using the two species of the parasite (*L. major* and *L. infantum*) showed that the three evaluated methods (agar dilution, cylinder plate and disk diffusion) can be effectively used for both species of parasite.

Cylinder plate and disk diffusion methods provided much better performance for preliminary evaluating of anti-leishmanial drugs in comparison with broth and agar dilution methods on promastigote stage. Cylinder plate and disk diffusion methods appeared equally acceptable for such susceptibility assay. The main drawback of the current study is that the assay has been set up for evaluating of promastigotes stage while the amastigotes are present in leishmaniasis patients. However such assays can be used for evaluation of amastigote like form of parasite (18).
